# Extended Inflammation Parameters (EIP) as Markers of Inflammation in Systemic Sclerosis

**DOI:** 10.1155/2024/3786206

**Published:** 2024-09-26

**Authors:** Anna Kowalska-Kępczyńska, Mateusz Mleczko, Kamila Komajda, Małgorzata Michalska-Jakubus, Dorota Krasowska, Maciej Korpysz

**Affiliations:** ^1^ Department of Biochemical Diagnostics Chair of Laboratory Diagnostics Medical University of Lublin, al. Solidarności 8, Lublin 20-081, Poland; ^2^ Department of Dermatology Venereology and Pediatric Dermatology Medical University of Lublin, ul. Staszica 11, Lublin 20-081, Poland; ^3^ Laboratory of Forensic Toxicology Medical University of Lublin, ul. Jaczewskiego 8b, Lublin 20-080, Poland

## Abstract

**Background:**

Systemic sclerosis (SSc) is an autoimmune disease characterized by inflammation, progressive vasculopathy, and fibrosis of skin and internal organs. The aim of the study was to evaluate extended inflammatory parameters (EIP) in patients with SSc in comparison to the control group of healthy subjects.

**Methods:**

A total of 28 patients with SSc and 29 healthy controls (HCs) were included in the study. The following EIP parameters were analyzed: neutrophil reactive intensity (NEUT-RI), neutrophil granularity intensity (NEUT-GI), antibody-synthesizing lymphocytes (AS-LYMP), and reactive lymphocytes (RE-LYMP).

**Results:**

Patients with SSc showed significantly higher values of parameters determining neutrophil reactivity and neutrophil granularity when compared to HCs (respectively, 49.16 FI vs. 44.33 FI, *p* < 0.001, and 152.01 SI vs. 147.51 SI, *p* < 0.001). Moreover, patients with SSc had higher absolute numbers of RE-LYMP than HCs (0.69 × 10^3^/*µ*l vs. 0.04 × 10^3^/*µ*l, *p* < 0.001). Importantly, significant correlations between the RE-LYMP and either IL-6 (*R* = 0.447, *p* < 0.001) or ESR (*R* = 0.532, *p* < 0.001) were found among patients with SSc.

**Conclusions:**

Changes in NEUT-RI, NEUT-GI, and RE-LYMP levels positively correlate with inflammation in SSc and, thus, could potentially be used as an additional reliable inflammatory biomarker to assess inflammation in this disease.

## 1. Introduction

Systemic sclerosis (SSc) is a multiorgan, autoimmune disease of connective tissue and vessels with an unclear etiopathogenesis. In genetically predisposed individuals, environmental factors are believed to stimulate a pathological cascade that leads to chronic systemic inflammation, progressive vasculopathy, and fibrosis of the skin and internal organs and ultimately to organ failure [1, 2]. The main events underlying the development of SSc include (1) endothelial cells (ECs) dysfunction, (2) dysregulation of the immune-inflammatory system with production of multiple cytokines and autoantibodies, and (3) transdifferentiation of fibroblasts to myofibroblasts with increased deposition of collagen and other extracellular matrix (ECM) compounds, which, together with dysregulated angiogenesis and defective vasculogenesis, cause fibroproliferative microvascular disorders with loss of capillaries [3].

**Figure 1:**
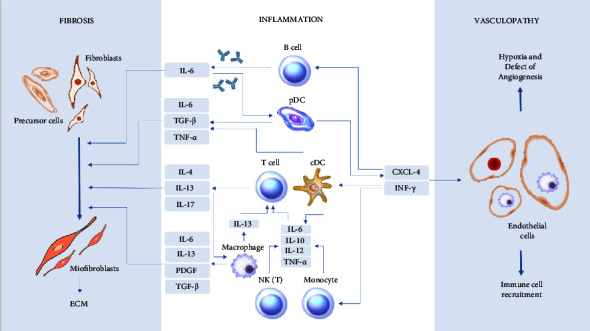
Role of immune cells in the pathogenesis of SSc. The figure shows three interconnected pathogenic processes in SSc: chronic systemic inflammation, progressive vasculopathy, and fibrosis of the skin and internal organs. cDC: conventional dendritic cells; CXCL-4: platelet factor 4; ECM: extracellular matrix; INF-*γ*: interferon *γ*; IL-4, IL-6, IL-10, IL-12, IL-13, and IL-17: interleukin-4, interleukin-6, interleukin-10, interleukin-12, interleukin-13, and interleukin-17; NK: natural killer cell; pDC: plasmacytoid dendritic cells; PDGF: platelet-derived growth factor; TNF-*α*: tumor necrosis factor-*α*; TGF-F062: transforming growth factor *β*.

Autoimmunity and inflammation are believed to be effectors of ECs damage as well as key factors influencing the profibrotic environment and, consequently, disease progression. Multiple cells and associated soluble mediators of both the innate and adaptive immune responses contribute to the initiation and amplification of inflammatory events in SSc. Several subsets of T helper (Th) cells, B cells, and myeloid cells have been reported to be increased or activated in the peripheral blood of SSc patients [4]. In recent years, other cell types, including neutrophils, have also attracted attention as players in SSc [5]. The role of immune cells in the pathogenesis of SSc is summarized in Figure 1.

Understanding the activation pathways of specific immune cells in SSc and disclosure of their biological markers may be the key to understanding the pathogenesis of the disease and assessing its activity, as well as an important step towards new personalized therapeutic strategies.

Among the indicators of immune system activation, innovative markers called extended inflammation parameters (EIP) are becoming more and more popular. These include neutrophil activation markers such as neutrophil-reactive intensity (NEUT-RI) and neutrophil granularity intensity (NEUT-GI) that are indicators of an early innate immune response, as well as the number of circulating reactive lymphocytes (RE-LYMP) and antibody-synthesizing lymphocytes (AS-LYMP), which are parameters for the quantitative assessment of activated lymphocytes. All these parameters are analyzed using fluorescence flow cytometry based on the cell size, intracellular structure, granules, and metabolic activity [6] and currently are integral parts of complete blood count analysis making them easily accessible in routine clinical practice. Specifically, RE-LYMP stands for reactive lymphocytes and reflects all lymphocytes that have a higher fluorescence signal than the normal lymphocyte population whereas AS-LYMP parameter quantifies the activated B lymphocytes (plasma cells) that synthesize antibodies. EIP values are amplified by intracellular proinflammatory pathways and depend on the type and intensity of the inflammatory stimulus [7, 8].

The EIP have never been reported in systemic sclerosis. Thus, the current study aimed to evaluate whether assessment of EIP may be a useful biomarker of inflammation in patients with SSc.

## 2. Materials and Methods

The study protocol was approved by the ethics committee of the Medical University of Lublin, obtaining consent for its implementation by resolution no. 23/2011. KE-0254/44/2021. Patients and healthy controls had signed an informed consent form. Privacy and confidentiality were maintained throughout the study process using a unique code number.

### 2.1. Characteristics of Study Population

The study group consisted of 28 patients (25 females and 3 males) over 18 years of age (mean age ± standard deviation [SD]: 60.0 ± 12.6 years) diagnosed with SSc based on the ACR/EULAR (American College of Radiology/The European Alliance of Associations for Rheumatology) 2013 classification criteria [9]. The control group consisted of age- and gender-matched healthy individuals, represented by 29 volunteers (21 females and 8 males) over the age of 18 years (mean age ± SD: 66.3 ± 15.5 years). The inclusion criteria of the study are age >18 years, diagnosis of SSc based on the ACR/EULAR 2013 classification criteria. The exclusion criteria of the study are uncontrolled concomitant chronic disease, cancer in the last 5 years, and pregnancy. The study population was recruited between September 2021 and November 2022 at the Department of Dermatology, Venereology and Pediatric Dermatology, Medical University of Lublin. Demographic and clinical parameters, including duration and subtype of the disease, and antinuclear antibodies (ANAs), were recorded.

Moreover, the study included laboratory parameters: C-reactive protein (CRP), erythrocyte sedimentation rate (ESR), and interleukin (IL)-6.

### 2.2. Materials and Methods

The research material was a venous blood collected in a 7.6 ml tube containing a coagulation activator and venous blood collected in a 2.7 ml tube with the addition of tripotassium ethylenediaminetetraacetic acid (K3EDTA). The material was used within 2 hours after collection.

A Sysmex XN 1500 flow cytometer was used to determine EIP parameters. EIP parameters were determined based on the assessment of cell fluorescence intensity and laser light scattering intensity, resulting from differences in the intracellular structure, granularity, and size of the analyzed cells. The following detailed EIP parameters were analyzed: NEUT-RI (neutrophil reactive intensity [FI]), NEUT-GI (neutrophil granularity intensity [SI]), AS-LYMP (antibody-synthesizing lymphocytes [10^3^/*µ*l]), and RE-LYMP (reactive lymphocytes [10^3^/*µ*l]).

In addition, “classic” biochemical parameters of inflammation were determined: ESR (erythrocyte sedimentation rate [mm/h]), CRP (C-reactive protein [mg/l]), and IL-6 (interleukin 6 [pg/ml]). Biochemical parameters were determined using the COBAS 6000 biochemical analyzer (Roche Diagnostics, Poland). ANAs titer was determined by indirect immunofluorescence using EUROIMMUN IIFT BIOCHIP technology. The titer was assumed as 0: negative (no antibodies), 1: low (<1 : 160), 2: average (1 : 160−1 : 320), and 3: high (>1 : 320).

### 2.3. Statistical Analysis

Statistical analysis was performed using Statistica 13.3 (1984–2017 TIBCO Software Inc) by StatSoft. The normality of the distribution of results obtained for individual parameters was checked using the Shapiro–Wilk test. Based on the obtained results, the statistical significance of the designated parameters was checked. For this purpose, the Student's *t*-test was used to compare groups with a normal distribution while maintaining the assumption of homogeneity of variances, and the Mann–Whitney test to compare groups with a nonnormal distribution. The analysis of qualitative variables was performed using the Kruskal–Walli's test and the chi-square test. A statistically significant level of *p* < 0.05 and a highly significant level of *p* < 0.001 were assumed.

## 3. Results

The demographic and clinical data of all study participants are shown in Table 1. In addition, in SSc group, the current treatment was recorded as follows: prednisone ≤10 mg/day (*n* = 4 patients), methylprednisolone ≤8 mg/day (*n* = 3 patients), methotrexate (*n* = 3), cyclophosphamide (*n* = 0 patients), mycophenolate mofetil (*n* = 4 patients), antimalarial drugs (*n* = 5 patients), nintedanib (*n* = 1 patient), sulodexide (*n* = 26 patients), pentoxifylline (*n* = 21 patients), prostaglandin E1 (*n* = 0 patients), and iloprost (*n* = 3 patients).

Among the analyzed laboratory parameters, SSc patients had a statistically significant increase in IL-6 levels (9.86 ± 20.41 vs. 2.16 ± 1.05; *p* < 0.001) in comparison to HCs.

### 3.1. Extended Inflammatory Parameters

The results of EIP parameters for all study participants are shown in Table 2. Among measured parameters, NEUT-RI and NEUT-GI showed statistically significant higher values in patients with SSc compared to HCs (152.01 SI vs. 147.57 SI, *p* < 0.001 and 49.16 FI vs. 44.33 FI, *p* < 0.001, respectively). Moreover, patients with SSc had higher absolute numbers of RE-LYMP compared to controls (0.69 × 10^3^/*µ*l vs. 0.04 × 10^3^/*µ*l, *p* < 0.001).

The results of EIP parameters among the SSc group according to the disease subtype and ANA titer are shown in Tables 3 and 4, respectively. There were no differences in EIP parameters depending on the SSc subtype and ANA titer in both groups.

**Figure 2:**
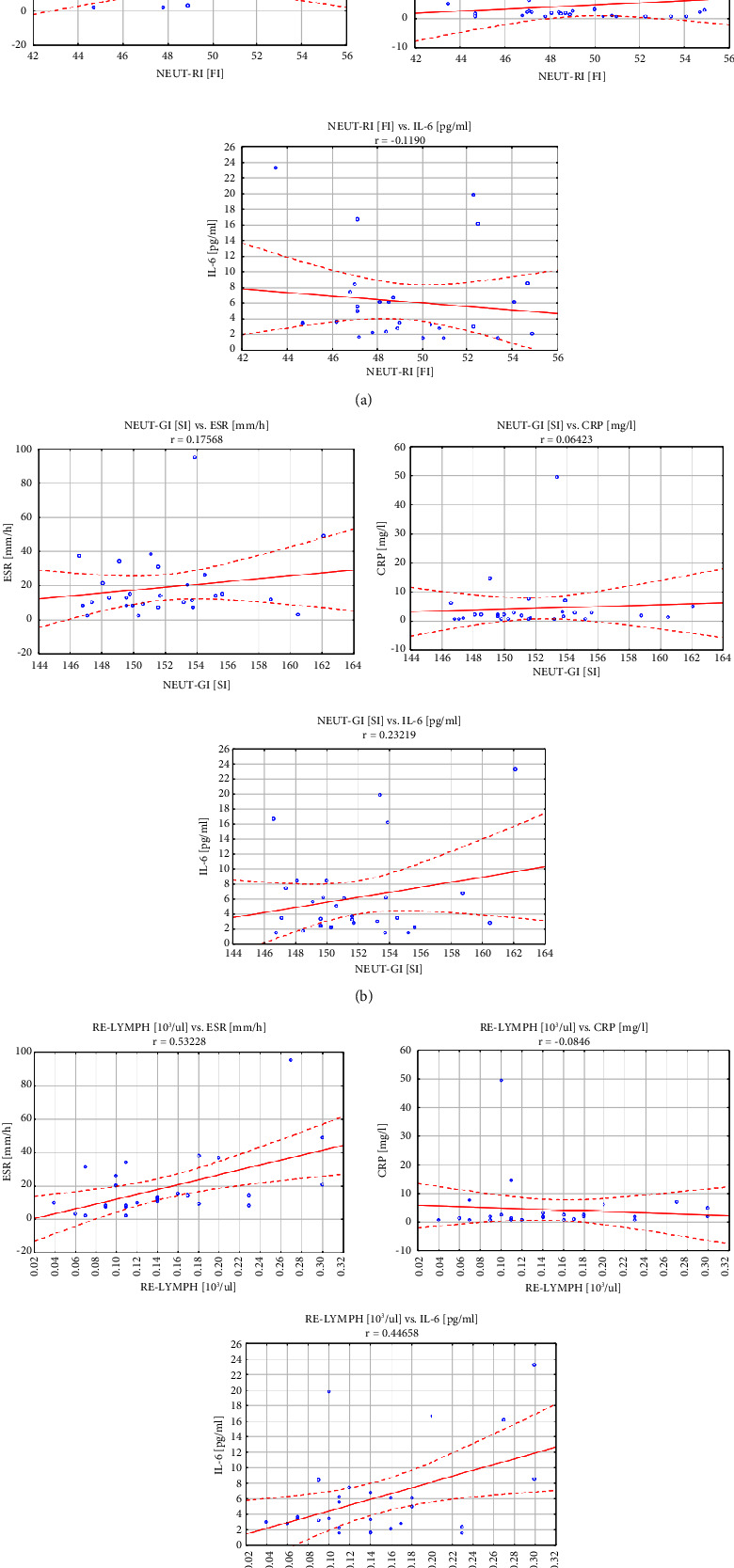
Correlation of NEUT-RI, NEUT-GI, RE-LYMP, and AS-LYMP parameters and CRP, ESR, and IL-6.

Moreover, associations of EIP parameters with other “classic” laboratory parameters of inflammation were found, including a significant positive correlation between the absolute number of RE-LYMP and the level of either ESR or IL-6. The correlation coefficients were 0.532 for the ESR level (*p* < 0.001) and 0.447 for the IL-6 level (*p* < 0.001). The remaining parameters from the EIP group did not show statistically significant correlations with any of the analyzed biochemical and clinical parameters. Correlations of NEUT-RI, NEUT-GI, RE-LYMP, and AS-LYMP parameters and CRP, ESR, and IL-6 have been shown in Figure 2.

## 4. Discussion

This is the first report to investigate the potential significance of EIP as markers of neutrophil and lymphocyte activation in systemic sclerosis (SSc). Dysregulated innate and adaptive immune responses driven by myeloid, B and T cells, are believed to be one of the key events in the disease pathogenesis. Autoimmune B-cell activation results in the production of autoantibodies directed against different nuclear self-antigens and several T helper (Th) cell subsets have been reported to be increased or activated in the peripheral blood of SSc patients as well as chronic mononuclear cell infiltration in affected areas [10–14]. What's more, neutrophils have been found to be engaged in the inflammatory process in systemic sclerosis [5, 15, 16]. Research is ongoing; however, there are still no good, easily accessible clinical biomarkers of the activation of the immune-inflammatory process in this disease. Therefore, there is a need to search for parameters that would allow the simple and quick, but reliable assessment of the functional activity of immune cells in SSc. To address this issue, in the present study, we analyzed quantitatively and compared EIP between SSc patients and healthy controls.

As a result, we showed significantly higher absolute numbers of reactive lymphocytes (RE-LYMP) in patients with SSc compared to healthy subjects, although there was no difference in the total number of lymphocytes between both groups (data not shown). The presence and percentage of RE-LYMP are thought to be crucial in indicating the inflammation process in the patient's blood as well as providing information about the activation of the immune response. Hence, our finding clearly points out an increase in activated lymphocytes in SSc, however, without accurate determination of cell subpopulations, since the RE-LYMP parameter reflects all lymphocytes that have a higher fluorescence signal than the normal lymphocyte population [17, 18]. In fact, considering the pathogenesis of systemic sclerosis, the increase in the number of activated lymphocytes is expected to be determined by both T and B lymphocytes [19, 20]. In current literature, among T cells, the type 2 T helper (TH2) cells, characterized by secretion of IL-4 and IL-13, regulatory T cells (Treg), and angiogenic T cells were observed to be increased in peripheral blood of SSc patients and to contribute to SSc development [19]. Obviously, increased numbers of reactive B lymphocytes and an evident B cell activation with the production of several autoantibodies have also been found in SSc patients in previous studies [19, 20]. Surprisingly, we could not find significant differences in AS-LYMP as separate parameter between SSc and control group as well as according to the titer of ANAs, probably due to small numerical values. Nonetheless, in the RE-LYMP count, the population of AS-LYMP that quantifies the activated B lymphocytes (plasma cells) synthesizing antibodies is always included [21]. Thus, increased RE-LYMP in SSc observed in our study may also partially stand for activation of the B-cell population.

The available literature reports the significant usefulness of the RE-LYMP parameter in the diagnosis and monitoring of inflammation in the course of viral infections [22], including the differentiation of SARS-CoV-2 (severe acute respiratory syndrome coronavirus 2) infection from bacterial infections [23] and autoimmune hepatitis (AIH) [24] in the course of which higher RE-LYMP values were observed in compared to a group of healthy people. The AS-LYMP parameter is considered important in the diagnosis of sepsis [25].

Noteworthy, in our previous research on psoriasis and pemphigus, we have shown that the number of activated lymphocytes expressed as the RE-LYMPH parameter perfectly reflects the advancement of inflammation in those patients during the autoimmune process [26, 27]. We showed that RE-LYMP values were significantly higher in the group of pemphigus patients compared to the control group, and a statistically significant increase in RE-LYMP values was observed depending on the extent of skin and/or mucous membrane lesions [26]. In people with psoriasis, we also observed an increase in RE-LYMP values compared to the group of healthy people, which correlates with the severity of the disease expressed in the PASI (Psoriasis Area and Severity Index) and BSA (Body Surface Area) scales [27].

The second new finding in the present study that should be emphasized is a positive correlation of RE-LYMP with “classic” descriptor of inflammation, i.e., ESR as well as IL-6. Such correlations have not been reported so far neither for noninfectious nor for infectious inflammatory diseases. The latter seems particularly promising since high levels of IL-6 in SSc were reported to correlate with the extent of skin involvement and with poor long-term outcomes [28]. Accordingly, our SSc patients had significantly increased levels of IL-6 compared to HCs, and this correlated with higher absolute numbers of reactive lymphocytes (RE-LYMP). IL-6 is a pleiotropic and proinflammatory cytokine that is produced among others by T and B cells, and it was identified as a factor mediating T-cell activation as well as B-cell stimulating cytokine that plays a crucial role in B-cell differentiation and immunoglobulin production [29]. Thus, the marked correlation of RE-LYMP with IL-6 levels in our SSc patients might suggest RE-LYMP as an easily accessible, reliable indicator of systemic inflammation in systemic sclerosis.

Finally, another important and pioneering disclosure of our study is a significant increase in parameters determining neutrophil's reactivity (NEUT-RI) and neutrophil's granularity (NEUT-GI) in patients with SSc compared to healthy subjects. Neutrophil granularity intensity (NEUT-GI) utilizes side-scattered light (SSC) to measure hypo- or hypergranulation, or dysplasia of neutrophil granulocytes, thus being an indicator of neutrophil phenotypic changes based on their internal structure. Changes in the intracellular structure of neutrophils may result from the mobilization of secretory vesicles and degranulation of neutrophil granules as a result of the action of proinflammatory cytokines [15, 30]. NEUT-GI increases in the presence of cytoplasmic granulation or vacuoles in activated neutrophils, reflecting primarily their phagocytic ability, intracellular enzyme content, and reactive oxygen species (ROS) production. Consequently, NEUT-GI reflects the increase in inflammatory processes [21, 30]. NEUT-RI is one of the neutrophil side-fluorescence light (NEUT-SFL) variables and reflects the metabolic activity of a neutrophil population. It increases in proportion to the quantity of nucleic acids in the cell providing information on DNA/RNA content. Both these parameters represent neutrophil activation and are indicators of an early innate immune response [17, 21, 30–32]. Importantly, neutrophils from SSc patients show signs of exaggerated activation but display several functional deficits, including disturbed phagocytosis, deficient myeloperoxidase (MPO) levels, and increased ROS generation that might contribute to inflammatory processes [5, 33, 34]. In fact, neutrophil-derived ROS have been found to be implicated in the pathogenesis of SSc by promoting endothelial cell dysfunction, fibrosis, and autoantigen response [16, 35, 36]. A dysregulated generation of neutrophil extracellular traps (NETs) and the process of NETosis (release of NETs) have also been described in SSc patients and may play a significant role in the disease pathogenesis by activation of the immune system against potential self-antigens [5, 15, 34, 35]. Aberrant NET generation appears to be closely linked with disease duration and the type of SSc-related complication, such as pulmonary arterial hypertension, digital ulcers, pulmonary interstitial disease, or extensive cutaneous fibrosis [35]. NETs are web-like structures composed of modified chromatin, histones, and proteins of neutrophil granules and cytoplasm, such as myeloperoxidase (MPO), and are considered as markers of neutrophil activation since activated neutrophils release NETs in response to a variety of stimuli [33, 34]. The study of Stiel et al. showed that the increase of NEU-SFL positively correlates with NET formation and hence could be considered as an indirect marker of NETosis [37]. Contrastingly, in other studies, levels of *ex vivo* NET production assessed by levels of DNA release negatively correlated with NEUT-RI, suggesting that change in neutrophil phenotype partially indicates their dysfunctionality and the secretion of NETs [38].

The available literature shows that the NEUT-RI and NEUT-GI parameters are very important descriptors in the course of inflammatory diseases. Higher values of these parameters were observed in patients with AIH compared to healthy people, which indicates their diagnostic potential [24]. It has been proven that the NEUT-RI parameter increases significantly in the course of sepsis and can be used to monitor it [25, 39]. Our previous studies also confirm the importance of the NEUT-RI and NEUT-GI parameters. It was shown that in patients with pemphigus (regardless of its type) [26] and in patients with psoriasis [27], the values of these parameters were significantly higher compared to healthy people.

**Figure 3:**
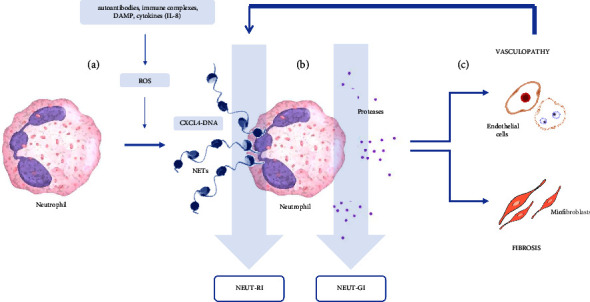
The importance of NEUT-RI and NEUT-GI parameters in the context of the pathogenesis of systemic sclerosis. (a) Different stimuli trigger neutrophil activation with increased ROS. (b) Increased degranulation of neutrophils and NETs release. (c) The content of neutrophil granules and components of NETs influence on the development of endothelial dysfunction and fibrosis. Measurement of the NEUT-GI parameter as an expression of cell phenotypic change and NEUT-RI as an expression of increased metabolic activity. DAMP: damage-associated molecular patterns; IL-8: interleukin-8; ROS: reactive oxygen species; CXCL4-DNA: complex of platelet factor 4 and DNA; NETs: neutrophil extracellular traps.

In line with this data, our findings of increased NEUT-RI and NEUT-GI indicate on marked neutrophil activation in SSc patients. The importance of NEUT-RI and NEUT-GI parameters in the context of the pathogenesis of systemic sclerosis has been shown in Figure 3.

## 5. Data Limitations

This study has potential limitations. The main limitations include (1) lack of correlation with clinical features of the disease, (2) lack of correlation with other markers of neutrophil activation, (3) small study group, and (4) laboratory and prelaboratory error.

## 6. Conclusions

The data presented in this study provide the first evidence that EIP parameters could potentially be used to assess inflammation in patients with systemic sclerosis.

Inflammatory process in SSc is driven by the activation of innate and adaptive immune cells, including lymphocytes and neutrophils. Activation of these cells is reflected by changes in their morphology. We have shown the presence of activated lymphocytes (increased RE-LYMP) and neutrophils (increased NEUT-RI and NEUT-GI) in the peripheral blood of patients with SSc by directly analyzing the white blood cell population using the automated cell analyzer. The increase correlated with well-grounded markers of inflammation such as ESR and IL-6. Hence, these results suggest that EIP obtained by flow cytometry may provide useful and rapid information on the status of the immune system and the degree of immune cell activation in the course of SSc available with routine morphology. However, in view of study limitations, the use of these new hematological parameters requires additional further research.

## Figures and Tables

**Table 1:** Demographic, clinical, and laboratory data of the study participants.

Variables	SSc patients (*n* = 28)	Control group (*n* = 29)	*P* value
*Demographic data*			
Age (years)			
Minimum-maximum	44–72	39–76	0.928
Mean ± SD	60.0 ± 12.6	66.3 ± 15.5	
Gender			
Women	25	21	0.075
Men	3	8	

*Clinical data*			
Disease duration (years), mean ± SD	11.1 ± 8.8	N/A	
Clinical subtype of SSc:			
lcSSc, *n* (%)	17 (60.7)	N/A	
dcSSc, *n* (%)	11 (39.3)		
ANAs:	28 (100)	2 (6.9)	<0.001
Low titer (<1 : 160), *n* (%)	7 (25)	2 (6.9)	
Average titer (1 : 160−1:320), *n* (%)	10 (35.7)	0 (0)	
High titer (>1 : 320), *n* (%)	11 (39.3)	0 (0)	
No, *n* (%)	0 (0)	27 (93.1)	

*Laboratory data*			
CRP (mg/l), mean ± SD	4.26 ± 9.18	2.72 ± 2.66	0.803
ESR (mm/h), mean ± SD	22.15 ± 21.86	10.35 ± 6.92	0.118
IL-6 (pg/ml), mean ± SD	9.86 ± 20.41	2.16 ± 1.05	<0.001

ANA, antinuclear antibody; CRP, C-reactive protein; dcSSc, diffuse cutaneous systemic sclerosis; ESR, erythrocyte sedimentation rate; IL-6, interleukin-6; lcSSc, limited cutaneous systemic sclerosis; SSc, systemic sclerosis; N/A, not applicable.

**Table 2:** Statistical characteristics of EIP parameters in patients with SSc and healthy controls (HCs).

Parameter	Group	M	Me	Min	Max	SD	*P* value
NEUT-RI (FI)	SSc patients	49.16	48.50	43.50	54.90	3.03	<0.001
	HCs	44.33	44.30	40.30	50.10	2.26	

NEUT-GI (SI)	SSc patients	152.01	151.60	146.60	162.10	3.92	<0.001
	HCs	147.57	146.90	140.40	157.00	4.13	

AS-LYMP (10^3^/*µ*l)	SSc patients	0.00	0.00	0.00	0.03	0.01	0.023
	HCs	0.00	0.00	0.00	0.00	0.00	

RE-LYMP (10^3^/*µ*l)	SSc patients	0.69	0.14	0.04	16.00	2.95	<0.001
	HCs	0.04	0.03	0.01	0.08	0.02	

AS-LYMP, antibody-synthesizing lymphocytes; M, mean; Max, maximum; Me, median; Min, minimum; NEUT-GI, neutrophil granularity intensity; NEUT-RI, neutrophil-reactive intensity; RE-LYMP, reactive lymphocytes; SD, standard deviation; SSc, systemic sclerosis.

**Table 3:** EIP parameters according to the SSc subtype.

Parameter	SSc subtype	M	Me	Min.	Max.	SD	H/Chi^2^	*P* value
NEUT-RI (FI)	lcSSc	48.99	48.70	43.50	54.70	3.03	1.762	0.414
	dcSSc	48.41	47.95	44.70	54.10	2.81		

NEUT-GI (SI)	lcSSc	153.24	153.70	146.60	162.10	4.31	4.227	0.121
	dcSSc	149.61	149.90	147.10	151.60	1.61		

AS-LYMP (10^3^/*µ*l)	lcSSc	0.00	0.00	0.00	0.01	0.004	0.144	0.930
	dcSSc	0.01	0.00	0.00	0.03	0.01		

RE-LYMP (10^3^/*µ*l)	lcSSc	0.15	0.14	0.04	0.30	0.08	0.172	0.918
	dcSSc	2.11	0.12	0.07	16.00	5.61		

AS-LYMP, antibody-synthesizing lymphocytes; dcSSc, diffuse cutaneous systemic sclerosis; lcSSc, limited cutaneous systemic sclerosis; M, mean; Max, maximum; Me, median; Min, minimum; NEUT-GI, neutrophil granularity intensity; NEUT-RI, neutrophil-reactive intensity; RE-LYMP, reactive lymphocytes; SD, standard deviation; SSc, systemic sclerosis.

**Table 4:** EIP parameters according to the ANAs titer.

Parameter	ANAs titer^∗^	M	Me	Min.	Max.	SD	H/Chi^2^	*P*
NEUT-RI (FI)	0	52.30	52.30	52.30	52.30	0.00	3.177	0.365
	1	47.38	47.10	46.80	48.10	0.55		
	2	49.18	48.55	44.70	54.70	3.11		
	3	48.99	48.50	43.50	54.90	4.00		

NEUT-GI (SI)	0	153.20	153.20	153.20	153.20	0.00	3.388	0.336
	1	148.90	149.10	146.60	151.10	1.90		
	2	152.09	152.25	147.10	158.80	3.92		
	3	152.17	151.60	146.80	162.10	4.58		

AS-LYMP (10^3^/*µ*l)	0	0.00	0.00	0.00	0.00	0.00	2.880	0.410
	1	0.00	0.00	0.00	0.01	0.01		
	2	0.00	0.00	0.00	0.01	0.01		
	3	0.01	0.00	0.00	0.03	0.01		

RE-LYMP (10^3^/*µ*l)	0	0.04	0.04	0.04	0.04	0.00	2.843	0.416
	1	0.14	0.12	0.11	0.20	0.04		
	2	0.17	0.16	0.07	0.30	0.09		
	3	1.91	0.14	0.07	16.00	5.28		

ANAs, antinuclear antibodies; AS-LYMP, antibody-synthesizing lymphocytes; M, mean; Max, maximum; Me, median; Min, minimum; NEUT-GI, neutrophil granularity intensity; NEUT-RI, neutrophil-reactive intensity; RE-LYMP, reactive lymphocytes; SD, standard deviation; SSc, systemic sclerosis. ^∗^0, negative (no antibodies); 1, low level (<1 : 160); 2, average level (1 : 160−1:320); 3, high level (>1 : 320).

## Data Availability

The data used in the study are sensitive patient data, belonging to the Independent Public Clinical Hospital No. 1 in Lublin. For this reason, they can only be shared with the consent of the hospital. In this case, please contact the Hospital's Data Protection Officer or the Director of the Hospital for Medical Affairs (ul. Staszica 16, 20-081 Lublin, https://www.szpital@spsk1.lublin.pl).
